# Translation and psychometric evaluation of composite feedback-seeking behavior questionnaire among Iranian medical residents

**DOI:** 10.1186/s12909-024-05586-w

**Published:** 2024-05-29

**Authors:** Amin Hoseini Shavoun, Azim Mirzazadeh, Homa Kashani, Seyed Reza Raeeskarami, Roghayeh Gandomkar

**Affiliations:** 1 Department of Medical Education, School of Medicine, Tehran University of Medical Sciences, No. 57, Hojatdoust St., Keshavarz Blvd, Tehran, Iran; 2 Department of Internal Medicine, School of Medicine, Tehran University of Medical Sciences, Tehran, Iran; 3 Department of Research Methodology and Data Analysis, Institute for Environmental Research, Tehran University of Medical Sciences, Tehran, Iran; 4 Department of Pediatrics, Imam Khomeini Hospital Complex, Tehran University of Medical Sciences, Tehran, Iran; 5 Health Professions Education Research Center, Tehran University of Medical Sciences, Tehran, Iran

**Keywords:** Feedback-seeking behavior, Self-motives, Psychometrics, Persian

## Abstract

**Background:**

Proactively seeking feedback from clinical supervisors, peers or other healthcare professionals is a valuable mechanism for residents to obtain useful information about and improve their performance in clinical settings. Given the scant studies investigating the limited aspects of psychometrics properties of the feedback-seeking instruments in medical education, this study aimed to translate the feedback-seeking behavior scales (frequency of feedback-seeking, motives of feedback-seeking, and promotion of feedback-seeking by supervisors) into Persian and evaluate the psychometric properties of the composite questionnaire among medical residents at Tehran University of Medical Sciences in Iran.

**Methods:**

In this cross-sectional study, feedback-seeking behavior scales were translated through the forward–backward method, and its face validity and content validity were assessed by 10 medical residents and 18 experts. The test-retest reliability was evaluated by administering the questionnaire to 20 medical residents on two testing occasions. A convenience sample of 548 residents completed the questionnaire. Construct validity was examined by exploratory factor analysis and confirmatory factor analysis and concurrent validity was determined by Pearson’s correlation coefficient.

**Results:**

Content validity assessment showed that the CVR (0.66 to 0.99) and CVI (0.82 to 0.99) values for items and S-CVI values (0.88 to 0.99) for scales were satisfactory. The exploratory and confirmatory factor analysis demonstrated that the models were confirmed with eight items and two factors (explaining 70.98% of the total variance) for the frequency of feedback-seeking scale, with 16 items and four factors (explaining 73.22% of the total variance) for the motives of feedback seeking scale and with four items and one factor (explaining 69.46% of the total variance) for promotion of feedback-seeking by supervisors. AVE values greater than 0.5 and discriminant validity correlations significantly less than 1.0 demonstrated that the total scores of the composite feedback-seeking behavior questionnaire had a favorable fit and the questions could fit their respective factors, and the latent variables were distinct. We found positive and significant correlations between the three scales and their subscales.

**Conclusion:**

The results of the present study supported the validity and reliability of the Persian composite feedback-seeking behavior questionnaire for assessing feedback-seeking behaviors in medical residents. Applying the questionnaire in residency programs may enhance the quality of clinical education.

## Introduction

Feedback is -crucial for learning in the workplace of medical residency training [1]. Yet, residents highlight that they are receiving inadequate feedback [2]. Many barriers including time constraints, workplace culture, residents’ reactions, limited interaction between attending physicians and residents and quality of feedback have been identified to impede the provision of feedback in workplace residency training [3–6]. To address this challenge, proactively seeking feedback from clinical supervisors, peers or other healthcare professionals is recognized as a valuable mechanism for residents to obtain useful information about and improve their performance in clinical settings [7, 8].

A total of 46 medical universities provide residency programs in Iran. The annual admission is more than 4600 residents distributed in 27 specialties. The minimum work hour is determined by 50 h of daily work per week plus night shifts ranging from twelve 18-hour shifts per month for first-year residents to six 18-hour shifts per month for fourth-year residents which is significantly more than in other countries [9]. On the other hand, the overwhelming workload on clinical teachers has been documented in workplace teaching settings in Iran [10, 11]. These factors have affected the quality of clinical education including the delivery of feedback to residents [12]. For instance, research findings in Iran have highlighted that most residents receive feedback occasionally [13, 14]. In this context, proactively seeking feedback can be a valuable tool to address the gap and to improve the clinical education in Iranian residency training.

## Literature review

Feedback-seeking behavior is a multifactorial concept influenced by individual dispositions and contextual factors [15, 16]. Self-motives and the promotion of feedback-seeking by supervisors have been demonstrated to play a vital role in the frequency of feedback-seeking as individuals and situational factors, respectively [17–22]. Previous research has highlighted several motives influencing the feedback-seeking behaviors: self-improvement, self-validation, ego protection, and impression defense [18, 20, 23]. The self-improvement motive refers to the resident’s desire to seek feedback to improve their knowledge, skills, and performance. These residents are more likely to be engaged in challenging activities that improve their learning and performance. Residents subject to self-validation tend to obtain accurate information about the self and hence choose diagnostic activities that provide the opportunity for asking precise feedback. Residents with ego protection motives consider negative feedback to be likely more threatening and tend to ask for less feedback [15]. Finally, the impression defense motive alludes to consistency between one’s central self-concept and new self-relevant information. Residents with this motive generally ask for affirming feedback after a weak performance [23]. As a situational factor, the promotion of feedback-seeking by supervisors is defined as the extent to which the environment values learning and growth so that residents feel comfortable and actively seek feedback without fear of retribution or negative consequences [21, 24].

Several scales have been developed and tested in the field of organizational psychology and medical education to measure feedback-seeking behavior and the influencing factors. Bose and Gijselaers (2013) used three of these scales; the frequency of feedback seeking [15], the motives of feedback seeking [10, 25] and the promotion of feedback seeking by supervisors [21] in residency training [20]. Other studies used one or two of these scales in medical residents and reported moderate to high internal consistency values [7, 26].

The first step for assessing the feedback-seeking behavior of residents would be to ensure the validity and reliability of the instruments. Our search identified several studies on the development or adaptation, and psychometric evaluation of tools for feedback delivery in residency training or medical students in Iran [14, 27, 28]. To our knowledge, we could not find studies on the psychometric assessment of the Persian version of feedback-seeking behavior scales in our clinical context. The use of feedback-seeking behavior tools helps to identify critical issues to improve the quality of clinical education of medical residents in Iran.

Considering the importance of feedback-seeking behavior in promoting medical residents’ performance, and given the scant studies investigating the limited aspects of psychometrics properties of the scales in medical education and the lack of Persian version of the instruments, the present study aimed to translate the three scales of feedback-seeking behaviors into Persian and evaluate the psychometric properties of the composite questionnaire among Iranian medical residents.

## Methods

### Study design

We considered a cross-sectional research design for an instrument translation and adaption with psychometric evaluation.

### Setting

This study was conducted on medical residents at hospitals affiliated with the Tehran University of Medical Sciences in Iran. The university admits more than 500 residents every year. At the time of this study, 1963 residents were studying in different residency years in 24 specialties. Duration of training ranges from 3 to 5 years depending on the types of specialties. The residency program includes bedside rounds, mortality or morning reports, lecture-based classes, morning reports, etc., and workplace training throughout clinical service provision. Residents receive feedback more or less during these training events from attending physicians. Assessment is a combination of workplace-based assessment during rotations (mainly based on Global Rating Forms), annual knowledge-based examination and pre-board qualifying and board certification exams at the end of residency training. Providing feedback may occur only after workplace-based assessments.

## Measures

In the present study, we employed feedback-seeking behavior instrument used by Bose and Gijselaers (2013). Their questionnaire consisted of two parts: (1) demographic information and (2) three feedback-seeking behavior scales [20].

The frequency of feedback-seeking scale was first developed by Ashford (1986) consisting of two sub-scales, monitoring refers to asking for feedback directly and inquiry alludes to indirectly finding out the feedback of others. The internal consistency of the monitoring subscale measured by Cronbach’s alpha coefficient was reported 0.77 and the inter-item correlation of the inquiry subscale was 0.33 [15]. In the Bose and Gijselaers (2013) version, the scale consists of 6 items rated on a 5-point Likert scale ranging from (1 = never to 5 = almost always) [20].

Motives of feedback-seeking scale comprise four subscales: motives of self-improvement, motives of self-validation, motives of ego protection, and motives of impression defense. The self-improvement and self-validation subscales were proposed by Janssen and Prins (2007) to measure the feedback-seeking motivation of medical residents at the University of the Netherlands. Cronbach’s alpha was reported 0.73 for seeking self-improvement information, and 0.86 for seeking of self-validation information [25]. The ego protection and impression defense subscales were suggested by Tuckey et al. (2002) and were tested on government employees and psychology students in Australia They reported good factor structure and internal consistency for the subscales (alpha coefficients equal to 0.91 and 0.85, respectively) [16]. This scale in the Bose and Gijselaers (2013) version comprises 16 items organized scoring on a 5-point Likert scale ranging from (1 = strongly disagree, 5 = strongly agree) [20].

The promotion of feedback seeking by supervisors was developed by Steelman et al. (2004) and examined in company employees. They reported an internal consistency reliability of 0.85 and a test-retest reliability of 0.53 to 0.70. Confirmatory factor analysis confirmed the one-factor model [21]. The Promotion of feedback seeking by supervisors has 4 items with the first two questions being scored in reverse, using a 6-point Likert scale ranging from (1 = totally disagree; 6 = totally agree) [20].

## Procedures

### Phase 1: translation and cultural adaption

After obtaining the developers’ permission from the main developer of the scales by email, translation and cross-cultural adaptation were conducted using the World Health Organization (WHO) guideline [29]. The scales were translated from English to Persian by two independent translators whose primary language was Persian and who had sufficient experience and mastery in translating English texts. Each translated version was compared to the original scales and discrepancies were resolved after discussion with the research team. Consensus was reached through discussion and reconciliation. The scales were back-translated into English by two translators who were blind to the original English versions. Thereafter, the research team reviewed the English translation version and compared it with the original scales. Corrections were applied and final Persian versions were prepared.

### Phase 2: psychometric evaluation

#### Content and face validity

To improve item quality and ensure the face validity of the translated scales, a face-to-face cognitive interview was conducted with 10 medical residents to identify and correct unclear, complicated, or inappropriate items. In the next step, a panel of 18 experts (14 women, and 11 assistant professors, 4 associate professors, and 3 professors) with expertise in medical education, clinical education or learning psychology was asked to assess each item of the scales using importance (3-point scale; 1 = not necessary, 2 = useful but not necessary and 3 = necessary), relevance (4-point scale; 1 = not relevant, 2 = somewhat relevant, 3 = relevant, and 4 = completely relevant) and simplicity (4-point scale; 1 = not simple 2 = item need some revision 3 = simple but need minor revision 4 = very simple) criteria. Experts were also requested to provide their comments on the understandability of items and the format of scales. The content validity of scales was assessed using the content validity ratio (CVR) and content validity index (CVI) coefficients. The CVR was calculated based on the formula = (Ne- (N / 2)) / (N / 2) in which Ne is the number of experts who have chosen the necessary option, and N is the total number of experts. A value equal to or greater than 0.42 was considered acceptable [30]. The CVI was computed for each item (I-CVI) and the overall scale (S-CVI). The I-CVI is the proportion of items rated as either 3 or 4 for importance, relevance and simplicity. S-CVI was calculated as the average score of the I-CVI [31]. The items with CVI greater than 0.79 were retained and those with CVI between 0.7 and 0.79 were modified and maintained [32].

All necessary changes were made and the three final Persian scales were prepared as a single composite questionnaire including demographic information age, gender, specialty and years of residency, named the feedback-seeking behavior questionnaire.

#### Test-retest reliability

We administered the feedback-seeking behavior questionnaire to a convenience sample of 20 residents during 2 weeks intervals for assessing test-retest reliability using the intraclass correlation coefficient (ICC) for each scale and subscale. The ICC values greater than 0.6 indicated the desired consistency [33].

#### Construct and concurrent validity and internal consistency reliability

##### Participants

We recruited residents from all residency years of different specialties. To determine the sample size, the “10 times rule” was the method of choice. This rule, which is well suited for PLS-SEM, indicates a minimum of 10 samples for per item [34–36]. A convenient sample of 548, distributed in proportion to the number of residents in different specialties was selected which is well above the sample size recommended by the rule to avoid a low response rate. Residents who had spent at least six months of their residency were included.

##### Data collection

Data were collected between January and February 2019. Residents were approached by the first author in the hospital setting. After signing the written informed consent, they were asked to complete the composite questionnaire in the paper-pencil format. The participation was voluntary and no reward was provided. Participants who did not have time to complete the questionnaire at the time of delivery were asked to complete it within two weeks.

##### Data analysis

To determine construct validity, first, exploratory factor analysis (EFA) was performed in SPSS software to determine the factorial structure of the scales. The Kaiser–Meyer–Olkin (KMO) test and Bartlett’s test were used to check the sample adequacy and sphericity of the scales, respectively. A KMO value equal to or above 0.6 was acceptable [37, 38]. The data was extracted through the principal component analysis (PCA) with Oblimin rotation to identify the underlying factors. Eigenvalues above 1.0 were considered to determine the number of factors. The minimum factor loading was determined at 0.5 with no common factor loadings.

The construct validity, subsequently, was evaluated according to confirmatory factor analysis (CFA) Smart-PLS3. Item reliability indices, convergent validity and divergent validity were calculated to evaluate the fit of the measurement model. The item reliability was checked using three criteria: coefficients of factor loadings, composite reliability (CR) and Cronbach’s alpha (α) coefficient. In this study, coefficients of factor loadings of 0.4 for CR and α values of 0.7 or higher were considered acceptable. The average variance extracted (AVE) criterion was used to measure convergent validity which should be equal or higher than 0.5. Divergent validity was investigated using reciprocal cross-loadings and the Fornell-Larker criterion, with considering the value for the appropriateness of 0.5. Finally, significant t-values were used to fit the structural model [39].

To check concurrent validity, we calculated Pearson’s correlation coefficient of the frequency of feedback-seeking with motives of the feedback-seeking and the promotion of the feedback-seeking by supervisors’ subscales. A p-value of 0.05 was considered statistically significant.

## Results

Of 548 medical residents that we approached, 538 (response rate = 98%), completed the questionnaire. A total of 538 returned questionnaires were included for analysis as 10 were discarded over incompleteness. Table 1 indicates participants’ demographic characteristics. Residents were from 24 different medical specialties. A total number of 347 (64.5%) participants were females. Participants were distributed among residency years. The mean age of participants was 32.2 ± 4.9 years (range 26–57 years).

**Table 1:** Demographic characteristics of the participants (*N* = 538)

Variables	*N*	%
Specialty Pathology Orthopedics Urology Anesthesia Radiation oncology Forensic medicine Sports medicine Nuclear medicine Dermatology General surgery Neurosurgery Ophthalmology Internal medicine Neurology Radiology Psychiatry Obstetrics and Gynecology Emergency medicine Occupational medicine Infectious diseases Cardiovascular Otorhinolaryngology Family medicine Physical medicine and rehabilitation	21 21 13 26 15 6 8 18 29 25 15 36 72 14 33 23 43 14 16 24 39 11 6 10	3.9 3.9 2.4 4.8 2.8 1.1 1.5 3.3 5.4 4.6 2.8 6.7 13.4 2.6 6.1 4.3 8.0 2.6 3.0 4.5 7.2 2.0 1.1 1.9
Sex Female Male	347 191	64.5 35.5
Residency Year PGY-1 PGY-2 PGY-3 PGY-4	164 151 140 83	30.5 28.1 26.0 15.4

### Content and face validity

Based on the experts’ comments and cognitive interviews, two items of the frequency of feedback-seeking scale (items 1 and 3) were double-barreled and each was broken into two items. Therefore, the final version of the frequency of feedback-seeking consisted of eight items. We also changed the response options for the motives of feedback-seeking from a five-point scale to a six-point response option set to adjust with the promotion of feedback-seeking by supervisors’ response format.

Table 2 shows the CVR and CVI results of three scales. The findings indicate that CVR values for items were in the range of 0.66 to 0.99. I- CVI values were between 0.82 and 0.99 and each measure obtained an S-CVI of 0.88 to 0.99 for relevance, clarity and simplicity criteria, which indicate values greater than content validity standards [12].

**Table 2:** CVI and CVR index values of feedback-seeking behavior questionnaire

Item	CVR (*N* = 18, CVR_thresh_. = 0.42)	I-CVI					
		Relevance		Clarity		Simplicity	
Frequency of feedback-seeking							
M1	0.88	0.99		0.83		0.88	
M2	0.88	0.99		0.83		0.88	
M3	0.88	0.99		0.77		0.88	
M4	0.66	0.94		0.94		0.88	
M5	0.66	0.94		0.94		0.88	
I1	0.99	0.99		0.99		0.94	
I2	0.99	0.99		0.99		0.99	
I3	0.88	0.88		0.83		0.77	
			S-CVI = 96.37		S-CVI = 0.89		S-CVI = 88.75
**Motives of feedback-seeking**							
SI1	0.88	0.99		0.94		0.94	
SI 2	0.99	0.94		0.99		0.99	
SI 3	0.88	0.94		0.99		0.94	
SI 4	0.99	0.99		0.99		0.99	
SV1	0.88	0.99		0.94		0.99	
SV 2	0.77	0.99		0.99		0.99	
SV 3	0.77	0.99		0.99		0.99	
SV 4	0.99	0.99		0.99		0.99	
EP1	0.99	0.94		0.94		0.94	
EP 2	0.77	0.94		0.94		0.94	
EP 3	0.88	0.94		0.94		0.94	
EP 4	0.88	0.99		0.83		0.88	
ID1	0.77	0.94		0.94		0.88	
ID 2	0.88	0.99		0.99		0.94	
ID 3	0.99	0.88		0.88		0.94	
ID 4	0.88	0.99		0.99		0.94	
			S-CVI = 0.96		S-CVI = 0.95		S-CVI = 0.95
**Promotion of feedback-seeking by supervisors**							
PFSS1	0.88	0.88		0.99		0.99	
PFSS 2	0.99	0.94		0.99		0.99	
PFSS 3	0.99	0.99		0.99		0.99	
PFSS 4	0.88	0.99		0.99		0.99	
			S-CVI = 0.95		S-CVI = 0.99		S-CVI = 0.99

CVR, Content Validity Ratio; CVI, Content Validity Index; I-CVI, Item-level CVI; S-CVI, Scale-level CVI

M (Monitoring); I (Inquiry); SI (Self-improvement); SV (Self-validation); EP (Ego protection); ID (Impression defense); PFSS (Promotion of feedback-seeking by supervisors)

### Construct validity

The KMO result showed that the total number of samples was sufficient and Bartlett’s test results reached statistical significance (*p* = .001), highlighting the data being suitable for performing an EFA (Table 3).

**Table 3:** Bartlett’s sphericity test for feedback-seeking behavior questionnaire

Scale		Frequency of feedback-seeking	Motives of feedback-seeking	Promotion of feedback-seeking by supervisors
KMO		0.779	0.829	0.813
Bartlett’s Test of Sphericity	Approx. Chi-Square	2267.32	4659.52	692.69
	df	28	120	6
	Sig.	0.001	0.001	0.001

As shown in Table 4, the results of the EFA revealed a two-factor structure for the frequency of feedback-seeking, a four-factor structure for motives of feedback-seeking and the extraction of only a one-factor structure for the promotion of feedback-seeking by supervisors.

**Table 4:** EFA of feedback-seeking behavior questionnaire

Items	Score, mean (SD)	Factors			
		1	2	3	4
Frequency of feedback-seeking		Monitoring	Inquiry		
M1	3.6 (0.9)	**0.806**			
M2	3.6 (0.9)	**0.848**			
M3	4.0 (0.8)	**0.744**			
M4	3.6 (0.9)	**0.804**			
M5	3.3 (0.9)	**0.803**			
I1	2.7 (1.0)		**0.556**		
I2	2.6 (1.0)		**0.984**		
I3	2.6 (1.0)		**0.884**		
**Motives of feedback-seeking**		**Self-improvement**	**Self-validation**	**Ego protection**	**Impression defense**
SI1	4.9(0.8)	**0.841**			
SI2	4.9 (0.7)	**0.863**			
SI3	5.0 (0.7)	**0.868**			
SI4	5.0 (0.7)	**0.863**			
SV1	4.0 (1.0)		**0.784**		
SV2	4.7 (1.0)		**0.818**		
SV3	4.9 (0.8)		**0.663**		
SV4	4.7 (0.8)		**0.846**		
EP1	3.2 (1.3)			**0.742**	
EP2	3.0 (1.2)			**0.770**	
EP3	3.4 (1.2)			**0.799**	
EP4	3.3 (1.3)			**0.841**	
ID1	3.4 (1.2)				**0.935**
ID2	3.4 (1.3)				**0.867**
ID3	3.5 (1.3)				**0.934**
ID4	3.7 (1.1)				**0.485**
**Promotion of feedback-seeking by supervisors**					
PFSS1	2.9 (1.5)	**0.774**			
PFSS2	2.6 (1.6)	**0.874**			
PFSS3	3.1 (1.6)	**0.808**			
PFSS4	3.1 (1.2)	**0.874**			

*Note* Items are bolded per column to indicate the relevant factor in which they belong. *p > .05

The convergent validity of the feedback-seeking behavior scales and their subscales retained appropriate with AVE ranging from 0.64 to 0.81, which passed the suggested criterion of 0.5 (Table 5).

**Table 5:** Convergent validity of feedback-seeking behavior questionnaire

Scale and subscale	AVE	t-value
**Frequency of feedback-seeking**	0.76	48.23
Monitoring	0.72	79.62
Inquiry	0.69	18.80
**Motives of feedback-seeking**	0.64	0.64
Self-improvement	0.81	7.86
Self-validation	0.68	7.49
Ego protection	0.67	5.51
Impression defense	0.73	5.21
**Promotion of feedback seeking by supervisors**	0.69	83.68

In Cross-loadings for discriminant validity assessment, there was a high correlation between items of the same construct and a very weak correlation between items of a different construct (Table 6).

**Table 6:** Discriminant validity of feedback-seeking behavior questionnaire by the method of Cross- loading analysis

Items				
Frequency of feedback-seeking	Monitoring	Inquiry		
M1	**0.908**	0.280		
M2	**0.917**	0.314		
M3	**0.804**	0.280		
M4	**0.768**	0.275		
M5	**0.881**	0.375		
I1	0.345	**0.903**		
I2	0.217	**0.749**		
I3	0.175	**0.788**		
**Motives of feedback-seeking**	**Self-improvement**	**Self-validation**	**Ego protection**	**Impression defense**
SI1	**0.885**	0.487	0.122	-0.001
SI2	**0.923**	0.541	0.092	0.031
SI3	**0.905**	0.515	0.104	0.046
SI4	**0.889**	0.488	0.044	0.004
SV1	0.349	**0.719**	0.053	-0.008
SV2	0.494	**0.862**	0.035	0.006
SV3	0.500	**0.805**	0.098	0.041
SV4	0.508	**0.914**	0.074	-0.030
EP1	-0.112	-0.082	**-0.789**	-0.401
EP2	-0.095	-0.086	**-0.805**	-0.393
EP3	-0.002	-0.005	**-0.818**	-0.468
EP4	-0.109	-0.076	**-0.860**	-0.414
ID1	-0.029	-0.013	-0.471	**-0.970**
ID2	-0.047	0.007	-0.521	**-0.928**
ID3	-0.021	-0.010	-0.455	**-0.960**
ID4	0.076	0.016	-0.264	**-0.471**
**Promotion of feedback-seeking by supervisors**				
PFSS1	**0.774**			
PFSS2	**0.872**			
PFSS3	**0.807**			
PFSS4	**0.873**			

*Note* The bolded values represent the factor loadings for each construct and the cross-loading are those not bold for the same construct. * Threshold value > .05

Table 7 shows that all construct passes the Fornell-Larker criterion, indicating there is no discriminant validity issue.

**Table 7:** Discriminant validity of feedback-seeking behavior questionnaire by the method of Fornell-Larcker criterion

Construct	1	2	3	4	5	6	7
1. Monitoring	**0.85**	0.33					
2. Inquiry		**0.83**					
3. Self-improvement			**0.90**		0.10	0.02	
4. Self-validation			0.56	**0.82**	0.07	0.00	
5. Ego protection					**0.81**		
6. Impression defense					0.51	**0.85**	
7. Promotion of feedback seeking by supervisors							**0.83**

*Note* The bold values are the square root of the average variance extracted AVE values of the latent variables. * Threshold value > .05

### Concurrent validity

As indicated in Table 8, the frequency of the feedback-seeking scale showed significantly positive correlations with the motives of the feedback-seeking scale (*r* = .34, *p* < .01), and the promotion of feedback-seeking by supervisors scale (*r* = .14, *p* < .01). The frequency of the feedback-seeking scale showed significantly positive correlations with all subscales of motives of feedback-seeking.

**Table 8:** Correlations of the feedback-seeking behavior scales and subscales

Scale	Frequency of feedback-seeking	Monitoring	Inquiry	
**Motives of feedback-seeking**	0.34**	0.38**	0.21**	
Self-improvement	0.27**	0.30**	0.16**	
Self-validation	0.36**	0.41**	0.21**	
Ego protection	0.16**	0.17**	0.10*	
Impression defense	0.18**	0.20**	0.12**	
**Promotion of feedback-seeking by supervisors**	0.14**	0.33**	0.20**	

*p < .05. **p < .0.01

### Reliability

Table 9 depicts reliability of scales and their subscales using ICC, CR and α. The highest ICC (= 0.88) was related to the motives of the self-validation. The CR value ranged between 0.89 and 0.94. α coefficients for all scales and subscales was more than 0.7 indicating acceptable internal consistency for the questionnaire. The highest α (= 0.93) was related to the motives of the self-improvement.

**Table 9:** The reliability of feedback-seeking behavior questionnaire

Scale and subscale	Number Items	ICC	CR	α
**Frequency of feedback-seeking**	8	0.81	0.91	0.82
Monitoring	5	0.71	0.91	0.87
Inquiry	3	0.83	0.90	0.85
**Motives of feedback-seeking**	16	0.79	0.91	0.82
Self-improvement	4	0.87	0.94	0.93
Self-validation	4	0.88	0.89	0.84
Ego protection	4	0.82	0.89	0.83
Impression defense	4	0.83	0.91	0.86
**Promotion of feedback seeking by supervisors**	4	0.72	0.90	0.85

Intraclass correlation coefficient (ICC), Composite reliability (CR), Cronbach’s alpha (α)

## Discussion

This study aimed to translate the feedback-seeking behaviors questionnaire composed of three scales: the frequency of feedback seeking, the motives of feedback seeking and the promotion of feedback seeking by supervisors into Persian and evaluate its psychometric properties (face validity, content validity, construct validity, concurrent validity, and reliability) among Iranian medical residents. To the best of our knowledge, we could not find a similar study in medical education examining the psychometric properties of feedback-seeking behavior scales comprehensively. The findings of this study showed that the Persian feedback-seeking behavior questionnaire had good validity and reliability in examining the feedback-seeking behavior of medical residents.

In the translation phase, an attempt was made to result the feedback-seeking behavior scales to be a correct semantic reflection of the English version. Content validity assessment of the questionnaire showed that the CVR (0.66 to 0.99) and CVI (0.82 to 0.99) values for items and S-CVI values (0.88 to 0.99) for scales were satisfactory considering the recommended values for 18 expert members [29, 31]. Corrections based on the qualitative comments enhanced the clarity and simplicity of the items. As a result, two items of the frequency of feedback-seeking scale were broken, each into two items. The final frequency of feedback-seeking scale consisted of 8 items: 5 items for the inquiry subscale and 3 items for the monitoring subscale. Bose and Gijselaers (2013) translated these scales from English to German and the number of subscales’ items changed compared to the original ones, but they did not delineate the translation process or content and face validity results [20]. This changes in different languages and context shows that the context characteristics influence the frequency of feedback-seeking behavior and highlights the importance of examining the scales in different workplace settings [40].

The EFA and CFA results showed that the models were confirmed with eight items and two factors (explaining 70.98% of the total variance) for the frequency of feedback-seeking scale, with 16 items and four factors (explaining 73.22% of the total variance) for the motives of feedback seeking scale and with four items and one factor (explaining 69.46% of the total variance) for promotion of feedback-seeking by supervisors in medical residents. This result is similar to the original version of the latter instrument [15]. The findings are also comparable with research on feedback provision instruments. Aligned with these findings, Ilaghi et al. (2023) demonstrated that four factors of “attitude towards feedback”, “quality of feedback”, “perceived importance of feedback”, and “reaction to feedback” explained 63.72% of the total variance of the fifteen-item REFLECT (Residency Education Feedback Level Evaluation in Clinical Training) questionnaire [27]. However, Teunissen et al. (2009) used the perceived feedback benefits instruments in obstetrics–gynecology residents in the Netherlands and found that the amount of variance explained on the perceived feedback benefits variable was only 13% [7].

AVE values greater than 0.5 and discriminant validity correlations significantly less than 1.0 demonstrated that the total scores of the feedback-seeking behavior scales had a favorable fit and the questions were able to fit their respective factors, and the latent variables were distinct. Studies that proposed original feedback-seeking behavior scales developed these scales generally based on robust theories and did not examine the internal structure of the scales [15]. Other researchers used the scales in different cultures without examining the construct validity [20]. Few studies that evaluated the internal structure of the feedback-seeking motives scale were in line with the current research, so that the two-factor model for the frequency of feedback-seeking and a single-factor model for the promotion of feedback-seeking by supervisors have been confirmed [15, 21]. The present study extends the evidence confirming the factor structure of feedback-seeking behavior measures in medical education experimentally. Further research is recommended to assess the construct validity of an 8-item scale of frequency of feedback-seeking in other settings.

Concurrent validity findings showed that the frequency of feedback-seeking scale was positively and significantly correlated to motives of feedback-seeking (*r* = .34) and the promotion of feedback-seeking by supervisors (*r* = .14). Several studies have highlighted the relations between the frequency of feedback-seeking and the motives in medical education [7, 14, 20] and non-medical education [18, 25] context. Teunissen et al. (2009) reported that residents who perceive more feedback benefits state a higher frequency of both feedback inquiry and feedback monitoring. They also found that higher perceived feedback costs result in more feedback monitoring [7]. Our striking finding was the weak relation between the frequency of feedback-seeking and the supervisor support which undermines the effects of contextual factors compared to personal characteristics. More studies are recommended to tested these findings.

The reliability of the scales in terms of internal consistency was high, α = 0.93 was reported 0.82 for the frequency of feedback-seeking and motives of feedback-seeking, and 0.85 for the promotion of feedback-seeking by supervisors. The CR value ranged between 0.89 and 0.94. Test-retest reliability for all scales were above 0.70. Reliability, particularly internal consistency, of feedback-seeking scales were the most evaluated psychometric properties. Studies reported good reliability for the scales [20, 21, 25], yet our results indicated higher reliability, which may be due to our large sample size. In addition, perceived feedback benefits had a relatively low Cronbach alpha in obstetrics–gynecology residents in the Netherlands [7].

### Research strengths and limitations

The current study was conducted with a large sample size in various medical specialties at Tehran University of Medical Sciences. Replicating the study with residents from other medical schools in Iran would increase the generalizability of the current results. It is also recommended that the composite feedback-seeking behavior questionnaire be translated into other languages and evaluated in other residency programs and medical and other healthcare training to identify its utilization in various contexts. Even though the feedback-seeking behavior questionnaire fulfilled the essential psychometric criteria, evaluating other aspects of validity such as the predictive validity of the questionnaire to predict medical trainees’ performance outcomes is recommended in future studies. It is also suggested to measure the correlations of the questionnaire with other data sources like attending physicians’ report of resident’ feedback-seeking behavior. Feedback-seeking behavior is a multifactorial concept. In this study, we examined the self-motives from individual characteristics and the promotion of feedback-seeking from the environmental factors aspect. Examining other influential factors can also be a line of inquiry.

### Implications for residency training

Implementing the Persian composite feedback-seeking behavior questionnaire reveals that how and how often residents use the feedback-seeking behaviors and determines the nature of their motives. It also identifies to what extent the residency training environment is supportive of seeking feedback. This information could be used for improving individual feedback-seeking behavior by residents, designing educational interventions by program directors and encouraging to adoption of supportive techniques by attending physicians.

## Conclusion

Feedback-seeking behavior influences residents’ clinical performance and impacts patient safety. Evaluating Feedback-seeking behavior and its influential factors can provide a basis for improving patient care. The results of the present study supported the validity and reliability of three Persian composite feedback-seeking behavior questionnaire in assessing feedback-seeking behaviors in medical residents. Applying this instrument in residency programs may enhance the quality of the programs.

## Data Availability

The datasets used and analyzed during the current study are available from the corresponding author upon reasonable request.
